# The Architecture of Cognitive Vulnerability to Depressive Symptoms in Adolescence: A Longitudinal Network Analysis Study

**DOI:** 10.1007/s10802-020-00733-5

**Published:** 2020-11-28

**Authors:** Igor Marchetti, Patrick Pössel, Ernst H. W. Koster

**Affiliations:** 1 Department of Life Sciences, Psychology Unit, University of Trieste, Via Edoardo Weiss, 21, 34128 Trieste, Italy; 2 Department of Counseling and Human Development, University of Louisville, Louisville, USA; 3 Department of Experimental-Clinical and Health Psychology, Ghent University, Ghent, Belgium

**Keywords:** Vulnerability, Depression, Adolescence network analysis, Moderated network analysis, Clinical psychology

## Abstract

Rates of subclinical symptoms and full-blown depression significantly increase during adolescence. Hence, understanding how multiple cognitive risk factors are related to depression in adolescence is of major importance. For this purpose, we simultaneously considered multiple cognitive vulnerabilities, as proposed by three major cognitive theories for depression, namely Beck’s cognitive theory, hopelessness theory, and response style theory. In this four-wave study, we investigated the architecture, interplay, and stability of cognitive vulnerability mechanisms, depressive symptoms, and stressors in a large group of adolescents over a period of one year (n = 469; mean age = 15 years; 64% female). Network analysis was used to shed light on the structure of cognitive vulnerabilities in a data-driven fashion. Analyses revealed that different cognitive vulnerabilities were intertwined and automatic thoughts played the role of hub node in the network. Moreover, the interplay among cognitive vulnerabilities and depressive symptoms was already markedly stable in adolescence and did not change over a 12-month period. Finally, no evidence was found that cognitive vulnerabilities interacted with stressors, as proposed by diathesis-stress models. These findings advance our understanding of multiple cognitive risk factors for depression in adolescence.

**Electronic Supplementary Material:**

The online version of this article (10.1007/s10802-020-00733-5) contains supplementary material, which is available to authorized users.

## Introduction

Adolescent depression is considered a global health crisis (Patel, 2013), provided it is associated with poor psychosocial functioning, poor academic performance, and lower physical and mental health (Andersen & Teicher, 2008; Fombonne, Wostear, Cooper, Harrington, & Rutter, 2001, Zisook et al., 2007). Subclinical levels of depression are present in more than 20% of adolescents (Bertha & Balazs, 2013) and about 18% of individuals experience full-blown depression before turning 19 year (Lu, 2019). Hence, examining the cognitive vulnerabilities associated with depressive symptoms in adolescence is of particular relevance since this transition period is characterized by a marked increase of depressive symptoms compared with younger cohorts (Bufferd, Dougherty, Carlson, Rose, & Klein, 2012; Lu, 2019). Additionally, early onset of depressive symptoms is associated with more problematic outcomes in adulthood, such as major depression and suicide (Fergusson, Horwood, Ridder, & Beautrais, 2005). Finally, understanding mechanisms associated with depression in adolescence may provide useful insights for prevention, which is increasingly considered a major priority (Cuijpers, Beekman, & Reynolds, 2012; Patel, 2013).

A variety of cognitive theories have been developed to understand the onset, maintenance, and recurrence of depressive symptoms. The three major theories are the *cognitive theory* (Beck, 1976), the *hopelessness theory* (Abramson, Metalsky, & Alloy. 1989), and the *response styles theory* (Nolen-Hoeksema, Girgus, & Seligman, 1992). Although characterized by different features (see below), these three theories share important similarities. For instance, they all adopt a vulnerability-stress perspective, in that encountering stressors is deemed to be necessary to activate cognitive vulnerability and, in turn, trigger the onset of depressive symptoms. Moreover, they have usually been proposed as sequential theories (Alloy, Clements, & Kolden, 1985), with some cognitive vulnerabilities being directly linked to depressive symptoms (*proximal vulnerabilities*, i.e., automatic thoughts or brooding), while other vulnerabilities being linked to symptoms primarily via other mechanisms (*distal vulnerabilities*, i.e., dysfunctional attitudes or negative cognitive style) (Pössel & Knopf, 2011). We briefly describe these three major theories below.

Beck (1976) describes in his cognitive theory the onset and maintenance of depressive symptoms as being due to a cascade of cognitive vulnerabilities, such as dysfunctional attitudes, cognitive errors, the negative cognitive triad, and negative automatic thoughts. According to Beck (1976), dysfunctional attitudes are rigid and maladaptive assumptions that substantially alter and steer information processing, when activated by stressors. The influence of dysfunctional attitudes is then exerted through cognitive errors, such as catastrophizing, overgeneralization, and selective abstraction, which have people draw negative and unhelpful interpretations. This leads the person to generate a system of negative beliefs that is characterized by a negative view of themselves, their future, and their surrounding world (i.e., negative cognitive triad). As a result, negative automatic thoughts, namely thoughts that come rapidly and automatically to mind when a person is stressed or upset, dominate mental activity and eventually spur the development of the emotional, somatic, and motivational symptoms of depression.

Similarly to the cognitive theory, the authors of the hopelessness theory emphasize the importance of altered cognitive processes, but their focus is specifically on negative cognitive style (Abramson et al., 1989). Negative cognitive style refers to attributing a negative event (i.e., stressor) to stable and global causes and drawing negative inferences about the self-worth of the individual and the consequences of the stressor. This renders the individual often unable to resolve their issues which causes hopelessness (negative view of their future; Pössel & Thomas, 2011). Research shows that the habitual adoption of this type of causal attributions and inferences over time leads to depressive symptoms (Alloy et al., 2000).

Finally, according to the response styles theory (Nolen-Hoeksema et al. 1992), what determines the onset, maintenance, and severity of depressive symptoms is the cognitive response to negative mood. If an individual reacts to stressors with rumination, namely negative, repetitive, self-referential thinking, they are likely to develop depressive symptoms over time. Among the different forms of rumination, the most maladaptive type is brooding, which refers to passively focusing on symptoms of distress and on the meaning of these symptoms (Treynor, Gonzalez, & Nolen-Hoeksema, 2003). Solid research shows that brooding is strongly associated with concurrent and future depressive symptoms and suicide attempts (Nolen-Hoeksema, Wisco, & Lyubomirsky, 2008; Rogers & Joiner, 2017).

These three cognitive theories have been extensively tested in adult populations and rapidly became the dominant framework for understanding the onset and maintenance of depression (Beck & Haigh, 2014; Liu, Kleiman, Nestor, & Cheek, 2015; Nolen-Hoeksema et al., 2008). Yet, a number of important issues remain poorly investigated and understood.

First, only a handful of studies have investigated vulnerabilities from multiple cognitive theories simultaneously (Lam, Smith, Checkley, Rijsdijk, & Sham, 2003; Pössel & Knopf, 2011; Smith, Alloy, & Abramson, 2006), while exploring the integration between these theories is crucial. In fact, the development of an integrated model could shed light on how a certain mechanism influences and is influenced back by the other vulnerability factors, regardless of the theory that originally proposed it. In turn, this could not only help us gain a deeper understanding of how vulnerability to depression functions, but also strengthen our clinical interventions. In fact, therapeutic procedures often rely on a certain cognitive model in order to modify mechanisms proposed in another model without any theoretical justification (i.e., technical eclecticism; Lampropoulos, 2001). For instance, in the context of the Penn Resiliency Program (Gillham, Jaycox, Reivich, Seligman, & Silver, 1990), adolescents are trained to question their inferences (i.e., hopelessness theory), by evaluating their accuracy and generating alternative inferences, as proposed in Beck's cognitive model. Second, most of the studies were conducted in adult samples, rather than adolescents (for exceptions, see Pössel & Pittard, 2019; Winkeljohn Black & Pössel, 2015). This is unfortunate, as previous studies showed that the transition from adolescence to adulthood is characterized by complex patterns of symptoms change (Costello, Copeland, & Angold, 2011). Third, little is known about whether the interplay among cognitive vulnerabilities in adolescence is stable or if it is subject to change due to the transitory nature of this developmental phase (Hankin et al., 2009). While cognitive vulnerabilities are posited to be relatively stable after late childhood (Hankin & Abramson, 2002), some have emphasized that vulnerabilities for depressive symptoms may change with brain maturation in adolescence (Davey, Yücel, & Allen, 2008).

Network analysis represents an alternative and innovative avenue to investigate the interplay between vulnerabilities from multiple cognitive theories. Based on graph theory, statistical network analysis is an exploratory approach, which conceives of psychological phenomena as causal systems where the constituting elements mutually influence and reinforce each other (Borsboom & Cramer, 2013). Crucially, network analysis provides important benefits over other approaches. First, it is a bottom-up approach, which does not require strong prior assumptions about the phenomenon under investigation (van den Berg et al., 2020). Although this does not constitute an added value per se, this feature can turn out very helpful when modelling many elements with no strong theoretical predictions about interrelations between variables or when mutually exclusive models are reported in the literature. Second, this approach generates a specific hypothesis about the possible network of causal links (called edges) among the constituting elements (called nodes) of the system (Dalege, Borsboom, van Harreveld, Conner, & van der Maas, 2016). By doing so, network models can easily model feedback loops (i.e., cyclic models), which are likely present in the context of psychopathology (i.e., bidirectional influence between rumination and sad mood) (Borsboom & Cramer, 2013). Third, network analysis can unveil previously unknown links among factors as well as identify which elements may play the most central role in the network (van den Berg et al., 2020). In turn, these pieces of information can both inform our knowledge of the phenomenon under investigation and propose new targets for clinical interventions. It is also important to mention, however, that network analysis shows several limitations, among which the fact that it does not model random error, it usually generates hundreds of parameter, whose relevance is evaluated in a subjective fashion, and the information elaborated at the group-level may transfer to the individual-level to a limited extant (Forbes, Wright, Markon, & Krueger, 2017a, b).

Although traditionally used to investigate symptoms and attitudes (Borsboom & Cramer, 2013; Dalege et al. 2016), network analysis has recently been applied to unveil the interplay among psychological constructs (i.e., structural network analysis; Epskamp, Rhemtulla, & Borsboom, 2017; Guyon, Falissard, & Kop, 2017), such as vulnerabilities to depression in adults (Faelens, Hoorelbeke, Fried, De Raedt, & Koster, 2019) and in remitted depressed individuals before and after treatment (Hoorelbeke, Marchetti, De Schryver, & Koster, 2016; Hoorelbeke, Van den Bergh, Wichers, & Koster, 2019). In the context of adolescence, previous studies have adopted network analysis to decipher the interrelationship among depressive symptoms (Mullarkey, Marchetti, & Beevers, 2019), emotional and behavioral problems (Boschloo, Schoevers, van Borkulo, Borsboom, & Oldehinkel, 2016), and posttraumatic stress symptoms (Russell, Neill, Carrión, & Weems, 2017). Recently, Bernstein and colleagues (2019) applied network analysis to investigate the temporal relationship between specific components of cognitive style, as derived from the hopelessness theory, and depressive symptoms in adolescents. Interestingly, this study showed that the development of stable and global negative attributions predict future depressive symptoms. However, no study has so far tested the structure and longitudinal (in)stability of multiple cognitive vulnerabilities derived from different theories of depression, stressors, and depressive symptoms in adolescents using network analysis.

In our longitudinal network analysis study, we measured several cognitive vulnerabilities derived from the three cognitive theories described above along with depressive symptoms and stressors (i.e., stressful life events). We recruited a large sample of adolescents and assessed them four times (wave 1 to wave 4) across a period of one year (i.e., one assessment session every four months). By doing so, we could model the relationship among cognitive vulnerabilities, stress, and depressive symptoms at cross-sectional level, and also investigate the stability of their interplay longitudinally. To meet these goals and ensure the trustworthiness of our results, we performed standard network analysis along with stability and accuracy checks. Finally, for the first time to our knowledge, we investigated the possible moderating role of stressful events (i.e., vulnerability-stress perspective) in the context of moderated network analysis (Haslbeck, Borsboom, & Waldorp, 2019).

## Methods

### Participants

At baseline 469 adolescents (mean age = 15.3 ± 0.7 years; 64% female) were recruited from freshmen classes at a Midwestern, partially suburban, public high school (total school population = 1,700) in the United States. The sample was largely White (73.8%; followed by 14.3% Black, 5.7% Latino, 3.4% mixed race/ethnicity, 2.8% other). The school’s student body mostly consisted of middle class families, with about one third of the students eligible for free or reduced lunch. From the first (wave 1) to the fourth wave (wave 4), 78 adolescents (60.3% females) dropped out of the study or were excluded due to missing data. There were no differences between the dropouts and remaining adolescents in gender, *χ*^2^_(1)_ = 0.38, *p* = 0.54, or race/ethnicity, *χ*^2^_(4)_ = 2.38, *p* = 0.30. However, dropouts were significantly older, *t*_(89.92)_ = 4.5, *p* < 0.001, Cohen’s *d* = 0.63 and reported more depressive symptoms at t1 than the remaining adolescents, *t*_(105.57)_ = 4.44, *p* = < 0.001, Cohen’s *d* = 0.56. This sample was previously used in another study (Winkeljohn Black & Pössel, 2015).

### Measures

In our study, we measured depressive symptoms (CES-D), stressors (LEC), and the vulnerability mechanisms, as proposed by the three theories of cognitive vulnerability to depression. As for Beck’s cognitive theory, dysfunctional attitudes (DAS), cognitive errors (CNCEQ), cognitive triad (CTI-C), and automatic thoughts (ATQ) were measured. As for the hopelessness theory and the response styles theory, negative cognitive styles (ACSQ) and rumination (brooding) were measured, respectively. Details about the measures are presented below.

**Depressive Symptoms.** The Center for Epidemiological Studies – Depression Scale (CES-D; Radloff, 1977) is a 20-item (e.g., “During the past week, there were things that upset me that usually do not upset me”) instrument developed for the screening of the depressive symptoms. Each item was rated on a four-point Likert scale. The internal consistencies of CES-D scores in the current sample range from α = 0.90 to 0.92 (skewness = 0.01 to 0.08; kurtosis = -0.19 to -0.34).

**Stressors.** The Life Event Checklist (LEC; Masten, Neemann, & Andenas, 1994) consists of 50 items listing chronic or discrete life events that may happen to adolescents. For each item, participants were asked to report whether or not the event happened in their lives within the past four months. Higher scores representing more life events (skewness = 0.05 to 0.16; kurtosis = 0.28 to -0.45).

**Dysfunctional Attitudes (Beck’s Cognitive Theory).** The Dysfunctional Attitudes Scale (DAS; Weissman & Beck, 1978) consists of 40 items (e.g., “I should be happy all the time”) measuring depressive beliefs as described in Beck’s cognitive theory (1976). In the current study, the DAS was modified to increase the readability for adolescents (Garber, Weiss, & Shanley, 1993). Each item was rated on a seven-point Likert scale. The internal consistencies of the DAS scores range in this sample from α = 0.79 to 0.85 (skewness = 0.0 to 0.01; kurtosis = -0.17 to -0.18).

**Cognitive Errors (Beck’s Cognitive Theory).** The Children’s Negative Cognitive Error Questionnaire (CNCEQ; Leitenberg, Yost, & Carroll-Wilson, 1986) is a 24-item self-report measure of cognitive errors. Participants were presented with scenarios and assessed the likelihood of responding in a particular way (e.g., “You invite one of your friends to stay overnight at your home. Another of your friends finds out about it. You think, ‘S/he will be really mad at me for not asking him/her and will never want to be friends again’”). Adolescents rated how much they agreed with the items on a five-point scale. The internal consistencies of the CNCEQ in the sample range from α = 0.94 to 0.96 (skewness = 0.01 to 0.11; kurtosis = -0.21 to -0.40).

**Cognitive Triad (Beck’s Cognitive Theory).** The Cognitive Triad Inventory for Children (CTI-C; Kaslow, Stark, Printz, Livingston, & Tsai, 1992) is a 36-item self-report measure of the negative view of the self (e.g., “I can do a lot of things well”), world (e.g., “The world is a very hostile place”), and future (e.g., “There is nothing to look forward to in the years ahead”). Each dimension is measured with ten items. Six items were added as filler items. Adolescents rated their agreement with the items on a three-point scale. The internal consistencies of the CTI-C score range in the sample from α = 0.92 to 0.94 (skewness = 0.04 to 0.09; kurtosis = -0.26 to -0.36).

**Automatic Thoughts (Beck’s Cognitive Theory).** The Automatic Thoughts Questionnaire (ATQ; Kendall, Howard, & Hays, 1989) consists of 40 items, consisting of 10 items about positive thoughts (e.g., “I am proud of myself) and 30 items about negative thoughts (e.g., “I wish I were a better person”). All items were rated on a 5-point Likert scale. In this study, we only considered negative automatic thoughts. Internal consistency ranges from Cronbach’s α = 0.96 to 0.97 (skewness = 0.09 to 0.19; kurtosis = -0.38 to -0.50).

**Negative Cognitive Style (Hopelessness Theory).** The Adolescent Cognitive Style Questionnaire (ACSQ; Hankin & Abramson, 2002) measures attributions and inferences about causes, consequences, and the self in relation to negative events (Abramson et al., 1989). The ACSQ entails 12 hypothetical negative scenarios, for which adolescents were asked to write down one cause. Then adolescents evaluated the stability and globality of the cause (negative inferences about the causes of negative events). Next, adolescents evaluated the probability of possible negative consequences (negative inferences about consequences) and the degree to which the occurrence of the event implied that they themselves are flawed (negative inferences about the self). Each rating used a seven-point scale. In line with a recent psychometric study (Giuntoli et al., 2019), internality was not considered and the construct was deemed as unidimensional. The internal consistencies of the ACSQ scale range from α = 0.96 to 0.97 in the current study (skewness = 0.05 to 0.10; kurtosis = -0.29 to -0.38).

**Rumination (Response Styles Theory).** The Rumination Response Subscale of the Response Styles Questionnaire (RRS; Nolen-Hoeksema & Morrow, 1991) consists of 18 4-point Likert items measuring how often a participant engaged in various behaviors in response to depressed mood. Based on Treynor and colleagues’ (2003) factor analysis, we specifically focused on the RRS subscale *brooding* (e.g., “When I feel down, sad, or depressed, I think‚ why do I always react this way?”). The internal consistencies of brooding range in this sample from α = 0.65 to 0.70 (skewness = 0.12 to 0.21; kurtosis = -0.51 to -0.55).

Importantly, across all measures, higher scores indicate higher levels of the construct being assessed.

### Procedures

Invitations for participating in this study were mailed to parents of students enrolled at the selected high school. Upon parental consent, students were asked for their assent. Four assessments were conducted at four-month intervals in group sessions during school hours. The University of Louisville provided Institutional Review Board approval for our study.

### Statistical Analysis

In our study, we aimed to accomplish the following objectives, (i) network estimation, namely building a psychological network representing the relationships among depressive symptoms, stressors, and cognitive vulnerability mechanisms; (ii) network inference, namely interpreting the local characteristics of the network, such as strength, predictability, and relative importance; (iii) network stability, accuracy, and intranetwork testing, namely checking the trustworthiness and replicability of the network analysis results and performing intranetwork comparison; (iv) moderated network analysis, namely investigating whether the stressors moderate the relationship among variables of the network; (v) temporal network, namely investigating whether the network changes over time, across the four time points.

First, we estimated the psychological network (i.e., EBIC graphical LASSO network; Epskamp & Fried, 2018). In non-technical terms, we computed partial correlations between each pair of variables (called nodes), after controlling for the influence of all the other variables of the network. Moreover, the magnitude of each correlation (called edges) was adjusted according to the LASSO correction (for more details, see Epskamp & Fried, 2018). By following this estimation procedure, each edge indicates an associative link, which cannot be due to a third variable included in the network (i.e., conditional dependence). Blue edges indicate a positive relationship between two nodes, while red edges indicate a negative relationship. Given that the presence of missing data can substantially alter the network structure (Borsboom, 2017), listwise deletion within each wave was applied.

Second, we shed light on which nodes were the most connected with the other variables and, consequently, could play an important role in the network (Borsboom & Cramer, 2013, but see Dablander & Hinne, 2019). Moreover, we pinpointed the exact pattern of links between nodes, highlighting the differences in the magnitude of association between nodes. To do so, we relied on three well-known indices, such as strength, predictability, and relative importance. Strength refers to the sum of the absolute weights of the edge connecting the node to all other nodes. Predictability refers to how well a certain node is predicted by all its neighboring nodes (Haslbeck & Waldorp, 2018). In order to clarify the amount of variance of every variable explained by each predictor, we performed a relative importance analysis (Johnson, 2000), whose replicability was ensured with tenfold cross-validation (James, Witten, Hastie, & Tibshirani, 2013). Predictive model fit (R^2^_CV_), derived from cross-validation, was compared with actual model fit (R^2^), with small differences indicating high replicability.

Third, we evaluated the degree of accuracy and reliability of the network, by bootstrapping the estimation procedure 1000 times (Epskamp et al., 2018). By doing so, we could evaluate the degree of precision and stability of each edge and the strength index. The former goal was accomplished by both inspecting the confidence intervals (CIs) around each edge, with narrower 95%-CIs indicating greater precision, and computing the correlation stability coefficient (i.e., CS-coefficient), with CS-coefficient equal or above to 0.5 being set as threshold for high stability (Epskamp et al., 2018). CS-coefficient was also computed for the strength index. Furthermore, by relying on the same bootstrap procedure, we computed intranetwork statistical differences at level of both edges and strength.

Fourth, we investigated whether stress could be a moderator of the relationships between the nodes of the network (Haslbeck et al., 2019). In other words, we tested whether the type (i.e., positive vs. negative) and the magnitude of each edge could be a function of the amount of stress reported by the participants, as suggested by the vulnerability-stress model (Alloy et al., 1985).

Finally, we tested whether the network of depressive symptoms, stressors, and vulnerabilities changed across the four waves. Preliminarily, we evaluated the degree of similarity (i.e., Spearman’s correlation) of edge weights, strength values, and predictability values across the different time points. Then, we tested whether the global connectivity (i.e., absolute sum of the network edges) and the general structure (i.e., maximum absolute element-wise difference) of the network were a function of time (van Borkulo & Millner, 2016). The network comparison procedure relied on a 5000-permutation test. Finally, considering the exploratory nature of this study, Bonferroni-Holm correction was applied.

## Results

### Descriptive Statistics

Means and standard deviations of cognitive vulnerabilities, depressive symptoms, and stressors are reported in Table 1. Pearson’s correlations across the four waves are shown in Table S1.

**Table 1:** Means, standard deviations, and range scores across the four waves

Variable	*Wave 1*			*Wave 2*			*Wave 3*			*Wave 4*		
	*M*	*SD*	*Range*	*M*	*SD*	*Range*	*M*	*SD*	*Range*	*M*	*SD*	*Range*
CES-D	19.13	11.28	0–57	16.32	11.70	0–57	15.23	11.28	0–52	15.01	11.75	0–55
DAS	105.82	17.67	49–200	101.17	17.23	40–164	98.75	17.54	40–162	97.40	18.40	40–173
CNCEQ	54.97	20.05	23–120	55.23	21.51	24–120	52.93	21.28	24–120	52.91	21.50	24–119
CTI-C	18.78	13.51	0–58	14.40	11.20	0–53	12.77	10.78	0–50	13.24	10.87	0–56
ACSQ	137.22	57.01	48–336	133.05	55.43	48–336	127.08	53.77	48–294	129.24	55.38	48–332
ATQ	61.72	25.27	30–150	59.90	26.18	30–150	56.68	25.29	30–150	55.67	24.21	30–150
Brooding	2.16	0.75	1–4	2.11	0.75	1–4	2.06	0.77	1–4	1.98	0.72	0–46
LEC	10.06	8.12	0–50	9.18	8.06	0–50	8.02	7.76	0–45	8.29	8.46	

*Note. M* and *SD* are used to represent mean and standard deviation, respectively. *CES-D*: Center for Epidemiological Studies – Depression scale; *DAS*: Dysfunctional Attitudes Scale; *CNCEQ*: Children’s Negative Cognitive Error Questionnaire; *CTI-C*: Cognitive Triad Inventory for Children; *ACSQ*: Adolescent Cognitive Style Questionnaire; *ATQ*: Automatic Thoughts Questionnaire; *Brooding*: RRS brooding subscale; *LEC*: Life Event Checklist

### Network Estimation

**Fig. 1:**
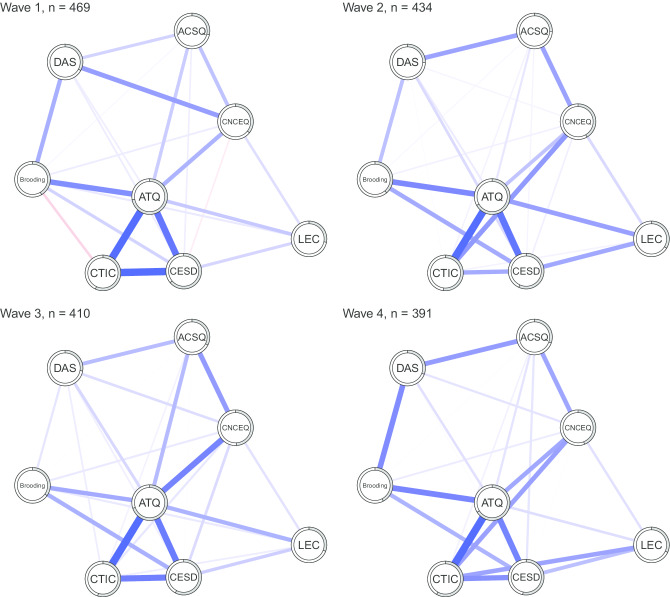
Network model across the four waves. *Note*: ACSQ = Adolescent Cognitive Style Questionnaire; ATQ = Automatic Thoughts Questionnaire; Brooding = brooding subscale of Response Styles Questionnaire; CES-D = Center for Epidemiological Studies – Depression Scale; CNCEQ = Children’s Negative Cognitive Error Questionnaire; CTIC = Cognitive Triad Inventory for Children; DAS = Dysfunctional Attitudes Scale; LEC = Life Event Checklist

Network models across the four waves are visualized in Fig. 1. The analysis revealed that the four networks were very similar to one another with respect to many features. First, although sparser after LASSO regularization, each network was characterized by a medium-to-high level of positive interconnectedness among the different nodes. This suggests the different mechanisms were not segregated, but linked with one another, despite their distinct theoretical backgrounds. Second, the node of automatic thoughts was highly connected to all the other variables included in the network, hence qualifying this cognitive vulnerability as highly central in the network (see network inference). Third, depressive symptoms were primarily connected to automatic thoughts, negative cognitive triad, brooding, and stressful events. Fourth, dysfunctional attitudes, negative cognitive style, and cognitive errors appeared to be more distal with respect to depressive symptoms and they were related to them primarily via other nodes, such as automatic thoughts and brooding. Fifth, the occurrence of stressful events impacted depressive symptoms both directly and indirectly, via automatic thoughts and negative cognitive triad.

### Network inference

**Fig. 2:**
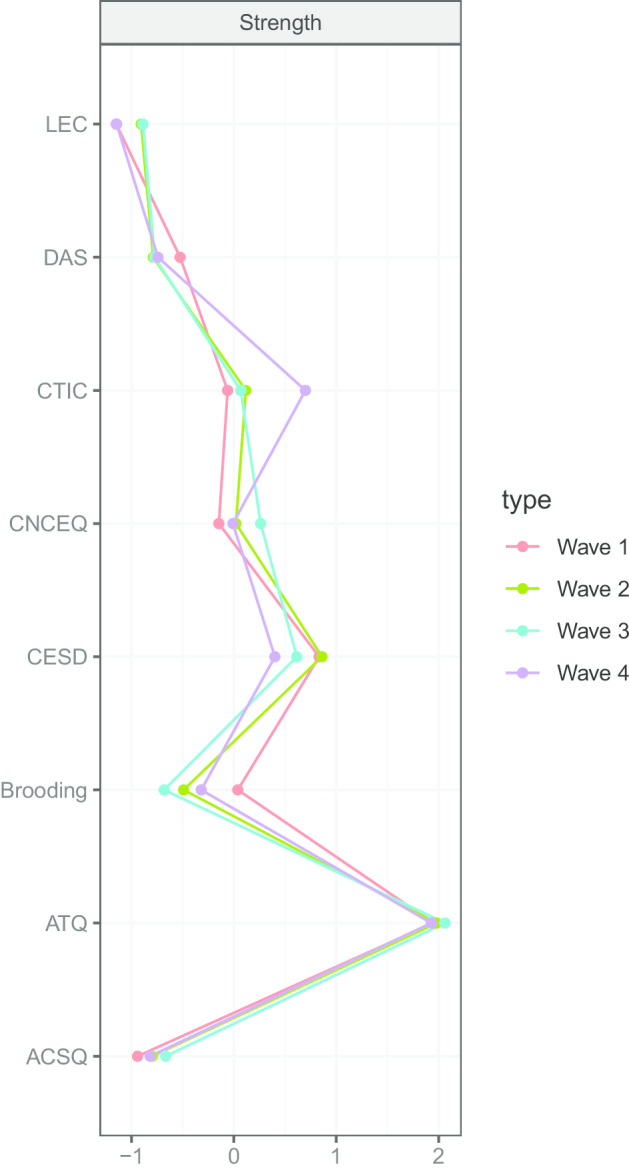
Strength scores, shown as standardized z scores, across the four waves

Network inference confirmed that automatic thoughts represented the strongest node, followed by depressive symptoms and negative cognitive triad (Fig. 2). This held very consistently across four waves. Dysfunctional attitudes, negative cognitive style, and stressful events were the least strong nodes in the network. Moreover, across the four waves, automatic thoughts emerged as the most predictable (*M*_*pred*_ = 0.69 ± 0.03), followed by depressive symptoms (*M*_*pred*_ = 0.58 ± 0.04), and negative cognitive triad (*M*_*pred*_ = 0.56 ± 0.03). The least predictable nodes were negative cognitive style (*M*_*pred*_ = 0.29 ± 0.03) and stressful events (*M*_*pred*_ = 0.31 ± 0.02). In other words, on average, 69% and 58% of variance of automatic thoughts and depressive symptoms could be explained by the other variables across the four waves, while only 29% and 31% of variance of negative cognitive style and stressful events could be accounted for. In order to clarify the amount of variance of depressive symptoms explained by each of the cognitive vulnerabilities and stressful events included in the network, we ran a relative importance analysis (Table 2). The analysis revealed that, across the four waves, automatic thoughts, cognitive triad, and brooding were the factors explaining the most variance of depressive symptoms, as compared with the other variables (Table S2). Importantly, the cross-validation analysis confirmed that the model fit is likely to be replicated in another sample, derived from the same population of adolescents.

**Table 2:** Relative importance analysis and cross-validation, with depressive symptoms being regressed on cognitive vulnerabilities and stressors

	Wave 1	Wave 2	Wave 3	Wave 4
DAS	3.74%	3.90%	3.97%	3.32%
CTI-C	20.19%	10.56%	12.60%	12.10%
CNCEQ	2.27%	4.91%	7.23%	5.10%
ATQ	21.01%	17.01%	17.01%	14.33%
Brooding	7.15%	8.38%	7.85%	7.75%
ACSQ	3.60%	2.67%	3.58%	3.88%
LEC	5.83%	8.19%	5.98%	6.14%
Model fit	R^2^ = 0.638	R^2^ = 0.556	R^2^ = 0.582	R^2^ = 0.526
Cross-validated model fit	R^2^_CV_ = 0.625	R^2^_CV_ = 0.538	R^2^_CV_ = 0.562	R^2^_CV_ = 0.518

*Note. DAS*: Dysfunctional Attitudes Scale; *CNCEQ*: Children’s Negative Cognitive Error Questionnaire; *CTI-C*: Cognitive Triad Inventory for Children; *ACSQ*: Adolescent Cognitive Style Questionnaire; *ATQ*: Automatic Thoughts Questionnaire; *Brooding*: RRS brooding subscale; *LEC*: Life Event Checklist

### Network Stability, Accuracy, and Intranetwork Testing

Stability analysis revealed that strength and edges were very stable, with *CS-coefficients* ranging from 0.67 to 0.75, across the four waves. In other words, even after dropping approximately 70% of the sample (up to 75%), a similar ranking of strength values and edges would be obtained in 95% of the cases. Moreover, accuracy check showed that both strength and edges were reliable and accurate (Figure S1 and S2), hence the reported networks were deemed valid and trustworthy.

Finally, across the four networks, automatic thoughts emerged as statically stronger than all the other nodes, followed by depressive symptoms (Figure S3). The edge difference test revealed that at least four edges were statistically different from the vast majority of the other edges, across the four networks, namely the edge between (i) automatic thoughts and depressive symptoms, (ii) automatic thoughts and negative cognitive triad, (iii) depressive symptoms and negative cognitive triad, and (iv) automatic thoughts and brooding (Figure S4).

### Moderated Network Analysis

The moderated network analysis estimated the network, by also including the possible moderating role of stressful events (Figure S5). Importantly, very similar results were obtained as compared to the networks reported in Fig. 1. For instance, automatic thoughts were again highly connected with the rest of the nodes, and depressive symptoms were mostly related to automatic thoughts, negative cognitive triad, brooding, and stressful events. Importantly, no significant interaction of stressful events with any pair of nodes was detected. In other words, across the four waves, stressful events did not moderate the link between any pair of variables in the network.

### Network Comparison

Our multi-step analysis for network comparison confirmed that the network did not change across the four waves, instead it remained remarkably stable over time (Table 3). First, the four networks had strongly similar edge weights (range r_s_ = 0.73 – 0.92), strength values (range r_s_ = 0.79 – 0.95), and predictability values (range r_s_ = 0.88 – 0.98). Moreover, formal tests showed that neither network global connectivity nor network structure changed during a one year period (all *p*’s > 0.88 and 0.07, respectively). In sum, converging evidence demonstrated that the network of cognitive vulnerabilities, depressive symptoms, and stressful events is already markedly stable in 15 year olds.

**Table 3:** Internetwork similarity indexes and network comparison tests across four waves

Comparison	Edge weights similarity	Strength similarity	Predictability similarity	Global connectivity difference	Network structure difference
Wave 1 – Wave 2	r_s_ = 0.71	r_s_ = 0.93	r_s_ = 0.90	*p* = 1	*p* = 0.07
Wave 1 – Wave 3	r_s_ = 0.82	r_s_ = 0.79	r_s_ = 0.90	*p* = 1	*p* = 1
Wave 1 – Wave 4	r_s_ = 0.75	r_s_ = 0.88	r_s_ = 0.88	*p* = 1	*p* = 0.32
Wave 2 – Wave 3	r_s_ = 0.88	r_s_ = 0.90	r_s_ = 0.93	*p* = 1	*p* = 1
Wave 2 – Wave 4	r_s_ = 0.92	r_s_ = 0.98	r_s_ = 0.98	*p* = 1	*p* = 1
Wave 3 – Wave 4	r_s_ = 0.83	r_s_ = 0.86	r_s_ = 0.90	*p* = 1	*p* = 1

*Note*. Spearman correlations (r_s_) were computed; *p*-values were adjusted for Bonferroni-Holm correction

### Additional Network Analyses

We further checked the trustworthiness of our analysis, by (i) replacing the CES-D with the Child Depression Inventory (CDI; Kovacs, 1992), (ii) excluding those items from the CES-D that were overlapping with the ATQ, and (iii) investigating the role of gender and age. When estimated with CDI, almost identical edge weights were obtained (range r_s_ = 0.92 – 0.96) (Figure S6 and S7). After removing items from the CES-D that were overlapping with the ATQ^1^, almost identical edges were obtained (range r_s_ = 0.93 – 0.97) (Figure S8 and S9). Moreover, when re-estimating the four networks after controlling for the impact of gender and age (Dalege et al., 2016), again, identical edge weights were obtained (range r_s_ = 0.95 – 0.99) (Figure S10 and S11).

Currently, there is no consensus as to how to deal with missing data in network analysis, with different methods being adopted (Bastiaanse et al., 2020). In order to take a cautious approach and in line with extant literature (Beard et al., 2016; Marchetti, 2019), we applied listwise deletion of missing data. However, we also checked the trustworthiness of our results, after imputing missing data via multivariate imputation by chained equations (MICE), as previously done (e.g., Levinson et al., 2018; Southward & Cheavens, 2018). It is important to stress that the networks estimated on listwise deleted or imputed data sets were almost identical in terms of edge weights (range r_s_ = 0.97 – 1), strength values (range r_s_ = 0.92 – 1), and predictability values (range r_s_ = 0.93 – 1). Imputed networks, strength values, and moderated networks are reported in the Supplementary Material (Figure S12, S13, and S14). Hence, no substantial difference at level of networks was found between the two types of missing data handling methods.

### Mean Levels Analysis

The analysis of the temporal unfolding of mean levels showed that all variables included in the model decreased over time. In detail, after applying the Bonferroni-Holm correction, there was a small temporal decrease of depressive symptoms (*F*_(3, 1170)_ = 14.2, *p* < 0.001, η^2^ = 0.011), dysfunctional attitudes (*F*_(3, 1170)_ = 31.9, *p* < 0.001, η^2^ = 0.028), negative cognitive triad (*F*_(3, 1170)_ = 35.9, *p* < 0.001, η^2^ = 0.036), cognitive errors (*F*_(3, 1170)_ = 3.48, *p* < 0.04, η^2^ = 0.003), automatic thoughts (*F*_(3, 1170)_ = 7.3, *p* < 0.0502 η^2^ = 0.006), brooding (*F*_(3, 1170)_ = 8.4, *p* < 0.001, η^2^ = 0.008), negative cognitive style (*F*_(3, 1170)_ = 7.9, *p* < 0.01, η^2^ = 0.006), and stressful events (*F*_(3, 1170)_ = 5.41 *p* < 0.01, η^2^ = 0.004). It is worth mentioning that, although significant, the reported reductions in mean levels were of negligible-to-small magnitude (i.e., η^2^ < 0.036).

## Discussion

Cognitive vulnerabilities to depressive symptoms in adolescents is increasingly attracting the attention of both scholars and clinicians, given that at least 27% of adults with major depression have had their first episode in adolescence (Kessler, Petukhova, Sampson, Zaslavsky, & Wittchen, 2012). Although many vulnerabilities have been identified, only limited efforts have been done to clarify how different vulnerabilities in concert lead to depressive symptoms. In our study, we adopted a network analysis approach to shed light on the specific interplay among major cognitive vulnerabilities in adolescence.

The analyses revealed that all the vulnerabilities considered were included in the network (i.e., no node was expelled), although they did not cluster in ways that were anticipated or specified in the original theories. For instance, negative cognitive style (hopelessness theory; Abramson et al., 1989) appeared to potentially exert an influence on depressive symptoms, mostly through automatic thoughts and cognitive errors (cognitive theory; Beck, 1976), while brooding (response styles theory; Nolen-Hoeksema et al., 1992) emerged to possibly play a mediating role between dysfunctional attitudes (cognitive theory) and depressive symptoms. Taken together, these findings clearly demonstrate that the vulnerabilities from the three original cognitive theories are related to each other and provide suggestions on how these cognitive vulnerabilities jointly impact depressive symptoms. Our study represents a preliminary step in the direction of a more comprehensive understanding of cognitive risk for depression.

Furthermore, our study also confirmed that the architecture of cognitive vulnerabilities to depressive symptoms is likely to be sequential (Alloy et al., 1985), with some cognitive vulnerabilities acting as proximal and others as distal vulnerabilities. Among the proximal cognitive vulnerabilities were automatic thoughts, the negative cognitive triad, and brooding, which could explain, across the four waves, about 17%, 14%, and 8% of depressive symptoms variance, respectively. In keeping with these results, previous studies have identified negative automatic thoughts as surface level cognitions (Kwon & Oei, 1994) that highly covary with depressive symptoms (Oei & Sullivan, 1999). Moreover, although Beck (1976) does not consider the negative cognitive triad as a direct contributor to depressive symptoms, but only through automatic thoughts, our study showed that having a negative view of the self, the future, and the world did have a direct relationship with depressive symptoms. This is in line with previous research showing that cognitive vulnerabilities impact depressive symptoms via both direct and indirect pathways (i.e., partial mediation) (Pössel & Winkeljohn Black, 2014). Finally, in accordance with the response style theory, brooding had a significant role in explaining depressive symptoms, although less than automatic thoughts and negative cognitive triad (i.e., 8% vs 17% and 14%, respectively).

Our study also highlighted three distal factors, namely dysfunctional attitudes, cognitive errors, and negative cognitive style, which were loosely connected with depressive symptoms. In keeping with the results of the network analysis, relative importance analysis showed that, when considered together, these distal vulnerabilities could account for only about 12% of depressive symptoms variance. It is perhaps worth mentioning that these cognitive vulnerabilities refer to cognitive contents and processes that are not expected to influence depressive symptoms directly, but via proximal factors. In fact, dysfunctional attitudes primarily refer to rigid and unrealistic attitudes about performance perfectionism and approval by others (i.e., cognitive content; Cane, Olinger, Gotlib, & Kuiper, 1986), while cognitive errors and negative cognitive style capture a biased way to elaborate negative information (i.e., cognitive process; Giuntoli et al., 2019). Hence, it is reasonable to presume that these distal vulnerabilities probably function at a deep level (Kwon & Oei, 1994) and their influence on depressive symptoms is primarily exerted through cognitive contents and processes that are expressed in the above mentioned proximal vulnerabilities, such as automatic thoughts, negative cognitive triad, and brooding.

Automatic thoughts emerged as the strongest node of the network and this result held very consistently across the four waves. Although it is tempting to equate node strength with causality, caution with this interpretation is recommended (Dablander & Hinne, 2019; Rodebaugh et al., 2018). In fact, on the one hand, in their seminal work Beck, Rush, Shaw, and Emery (1979) proposed that negative automatic thoughts act as mediator of change in cognitive therapy, hence implying that these cognitions play a causal role in eliciting depressive symptoms. On the other hand, a recent systematic review of the literature showed that only in about half of the studies the mediating role of the automatic thoughts was empirically confirmed (Lemmens, Müller, Arntz, & Huibers, 2016). This latter point raises the possibility that automatic thoughts may not always be the source of depressive symptoms, but in some instances reflect a by-product of other cognitive vulnerabilities (i.e., negative cognitive triad or brooding) or depressive symptoms per se. In the light of our study, future studies should clarify the causal status of the automatic thoughts by means of direct experimental manipulation (Lemmens et al., 2016).

To our knowledge for the first time in the context of network analysis, we tested whether experiencing stressful events could interact with cognitive vulnerabilities when impacting depressive symptoms (i.e., moderated network analysis). Although our analysis revealed no evidence supporting the vulnerability-stress model, we recommend caution in interpreting this result (“absence of evidence is not evidence of absence”; Altman & Bland, 1995), as many reasons, among which statistical, methodological, and theoretical ones, could account for this result.

First, our study may have failed to detect significant interactions due to power issues (i.e., Type II error). Although possible, this explanation is unlikely given that previous studies have shown that high level of true-positive rate (sensitivity) and true-negative rate (specificity) are reached in networks with features similar to ours (i.e., less than 26 nodes, normally distributed nodes, gamma parameter equal to 0.5; Epskamp, 2016). Second, we only evaluated the occurrence of external stressors (e.g., arguments with the parents or receiving a failing grade), while internal stressors of mental (i.e., spontaneous thoughts, feelings, and mood; Beck et al., 1979; Marchetti, Koster, Koster, & Alloy, 2016) or organic (i.e., neuro-inflammation; Maes et al., 2009) nature were not considered. Third, not all the theories considered in this study attribute the same importance to the role of stressful events. For instance, the role of stressors is central for the activation of the vulnerability factors for the hopelessness theory and for some of the vulnerability factors in Beck’s cognitive theory, while their role is less vital for the response styles theory. Moreover, cognitive theories often stress that the individual’s vulnerability is reactive only to specific types of stressors (i.e., sociotropy and interpersonal stressors; Hammen, Ellicott, Gitlin, & Jaminson, 1989). In our study, we did not consider stress-vulnerability type matching. Fourth, we only considered contemporaneous networks in our study, an aspect which might have obscured the joint impact of stressful events and vulnerability factors on depressive symptoms over time (i.e., temporal networks; Epskamp et al., 2018). However, at the theoretical level, our results may also be interpreted as being in line with the alternative etiologies of cognitive vulnerabilities (Parry & Brewin, 1988). According to this model, no interaction between stressors and vulnerabilities is required, as they both impact depressive symptoms, but in an independent fashion. In sum, although our moderated network analysis did not provide any evidence supporting the diathesis-stress approach in adolescence, many interpretations could be proposed. Hence, future studies should further explore this crucial aspect of vulnerability to depression and depressive symptoms with appropriate research designs and more sensitive measures for both stressors and vulnerabilities.

Finally, our study revealed that the interrelationship among cognitive vulnerabilities, depressive symptoms, and stressors is markedly stable at about 15 years old, in that no difference in the network structure was detected at this age during a period of 12 months. Our findings are in line with previous research (Hankin et al., 2009), showing that already during middle adolescence (i.e., 15–17 years old) cognitive vulnerabilities are somewhat stable as compared to childhood or early adolescence (Hankin, 2008; Romens, Abramson, & Alloy, 2009). Moreover, again in line with previous research (Hankin et al., 2009), small-to-negligible reductions in the mean levels of the constructs investigated were found. Although difficult to explain, these fluctuations have been usually attributed to suboptimal psychometric properties of the instruments and regression toward the mean (Hankin, 2008; Romens et al., 2009). Overall, our study suggested that network structure and intensity of cognitive vulnerabilities and depressive symptoms has reached a point of marked stability already in middle adolescence, and perhaps even earlier.

Our study is characterized by both strengths and limitations. Among the former is that, first, we investigated the interrelationship among several cognitive vulnerabilities by relying on network analysis. Although previous studies have attempted an integration of the cognitive theories of depressive vulnerability (Lam et al., 2003; Pössel & Knopf, 2011; Smith et al., 2006), different and sometimes mutually exclusive models have been proposed in the literature. Hence, considering that there is no consensus as to how the different mechanisms are interrelated, in our study we did not impose any a-priori constraint on the statistical model and took full advantage of the exploratory nature of network analysis. We recommend that future studies complement our results with confirmatory techniques, such as structural equation modeling. Second, in this study, we considered the major cognitive vulnerabilities as described in the three major cognitive theories of depression, and we followed them up in a longitudinal manner. Third, we ran sophisticated analyses, such as moderated network analysis and cross-validation analysis, in order to increase the informativeness and replicability of our findings. Fourth, our results held, even after changing the measure of depressive symptoms (i.e., from CES-D to CDI), hence implying a high degree of trustworthiness.

Among the limitations is, first, the sole reliance on self-report questionnaires. While different methods are available for capturing depressive symptoms and life stressors, only self-report questionnaires are available for measuring cognitive vulnerabilities (Gotlib & Neubauer, 2000). Hence, we preferred relying on the same method, in order to avoid discrepancy among the different nodes of the network. Second, we only followed the adolescents for a total period of about 12 months, which might have prevented detecting changes in the network structure that require a longer period to emerge. It is worth mentioning, however, that substantial stability of cognitive vulnerabilities was also found in a 7-year longitudinal study on adolescents (Romens et al., 2009). Third, it is not clear to what extent our sample was representative of the students at the school, where this study was carried out, and of the general adolescent population. Future studies may want to consider a more stratified recruitment to ensure as much as possible that participants were demographically representative of the student population. Moreover, about 16% of participants dropped out across the four waves. Although not negligible, the attrition rate is in line with previous studies carried out in adolescents (i.e., Hankin, 2008) and, importantly, almost identical results were obtained when missing data was treated with imputation procedure. Fourth, given the presence of only four timepoints, it was not possible to estimate temporal networks, such as graphical vector autoregressive modelling (Epskamp, Waldorp, Mõttus, & Borsboom, 2018) and cross‐lagged panel network (Rhemtulla, van Bork, & Cramer, 2017). Future studies could directly address this important research question, by implementing longitudinal studies with multiple assessment points over the period of one year or more.

Our study paves the way for future studies. For instance, although several cognitive vulnerabilities were considered simultaneously, only about 57% of the variance of depressive symptoms across the four waves could be accounted for. Note that this estimate is likely to be inflated, as it is highly unlikely that all the cognitive vulnerabilities investigated actually exert a causal influence on depressive symptoms. Hence, future studies should consider adopting a multilevel and multifactor approach (Hankin et al., 2009), where other crucial mechanisms are included, such as cognitive biases (Marchetti et al., 2018), temperament (Compas, Connor-Smith, & Jaser, 2004), social support from families, friends, and teachers (Pössel et al., 2018), neural pattern (Davey et al., 2008), and genetic substrate (Fox & Beevers, 2016). Furthermore, our study was silent with respect to the temporal dynamics that generated the network of cognitive vulnerabilities and their trajectories after adolescence.

The findings of this study have important implications, in that they could help calibrate clinical interventions in the context of depressive risk in adolescents. Our results indeed suggest that, on the one hand, targeting automatic thoughts, cognitive triad, and brooding may reduce depressive symptoms in an effective way. Interestingly, this datum spurs for the integration of techniques derived from different theories, such as the “ABC” technique for detecting and changing automatic thoughts (Beck’s cognitive model; Beck et al., 1979), the cognitive restructuring for modifying the cognitive triad (Beck’s cognitive model; Beck et al., 1979), and the rumination-focused cognitive therapy for reducing brooding (response style theory; Watkins, 2018). On the other hand, clinical or experimental interventions on inferences, dysfunctional attitudes, and cognitive errors could be helpful in hindering the onset and recurrence, but they could have limited impact on depressive symptoms per se. In sum, our study provides an emerging framework for guiding clinicians in their work with adolescents with depressive symptoms.

In conclusion, our study shows that, at the age of 15 years old, the architecture of cognitive vulnerability is already markedly stable, with some vulnerabilities being more central and proximal (i.e., automatic thoughts, cognitive triad, and brooding) and others being more distal (i.e., negative cognitive style, dysfunctional attitudes, and cognitive errors).

## Electronic Supplementary Material

Below is the link to the electronic supplementary material.

Supplementary file1 (DOCX 2.41 MB)
