# Supplementary material for: The Architecture of Cognitive Vulnerability to Depressive Symptoms in Adolescence: A Longitudinal Network Analysis Study
Source: Res Child Adolesc Psychopathol. 2020 Nov 28;49(2):267–81. doi: 10.1007/s10802-020-00733-5 (PMC7826312; doi:10.1007/s10802-020-00733-5)
Supplement: Supplementary file 1 — Supplementary file1 (DOCX 2.41 MB) [file 10802_2020_733_MOESM1_ESM.docx]

**Supplementary Material**

**Table S1.** *Pearson’s correlations among vulnerabilities, depressive symptoms, and stressors across the four waves*

|  | t1_CESD | t1_ DAS | t1_CTI-C | t1_CNCEQ | t1_ATQ | t1_brooding | t1_ACSQ | t1_LEC | t2_CESD | t2_das | t2_CTI-C | t2_CNCEQ | t2_ATQ | t2_brooding | t2_ACSQ | t2_LEC |
| --- | --- | --- | --- | --- | --- | --- | --- | --- | --- | --- | --- | --- | --- | --- | --- | --- |
| t1_CESD | 1 | 0.41 | 0.7 | 0.34 | 0.76 | 0.5 | 0.4 | 0.42 | 0.53 | 0.3 | 0.36 | 0.29 | 0.45 | 0.34 | 0.2 | 1 |
| t1_DAS | 0.41 | 1 | 0.29 | 0.43 | 0.49 | 0.46 | 0.41 | 0.31 | 0.38 | 0.5 | 0.28 | 0.4 | 0.37 | 0.38 | 0.27 | 0.41 |
| t1_CTI-C | 0.7 | 0.29 | 1 | 0.33 | 0.68 | 0.29 | 0.35 | 0.32 | 0.32 | 0.2 | 0.36 | 0.25 | 0.32 | 0.18 | 0.12 | 0.7 |
| t1_CNCEQ | 0.34 | 0.43 | 0.33 | 1 | 0.51 | 0.38 | 0.41 | 0.34 | 0.49 | 0.34 | 0.45 | 0.68 | 0.5 | 0.38 | 0.33 | 0.34 |
| t1_ATQ | 0.76 | 0.49 | 0.68 | 0.51 | 1 | 0.57 | 0.49 | 0.46 | 0.52 | 0.35 | 0.46 | 0.41 | 0.57 | 0.4 | 0.28 | 0.76 |
| t1_brooding | 0.5 | 0.46 | 0.29 | 0.38 | 0.57 | 1 | 0.34 | 0.33 | 0.43 | 0.31 | 0.29 | 0.36 | 0.43 | 0.57 | 0.24 | 0.5 |
| t1_ACSQ | 0.4 | 0.41 | 0.35 | 0.41 | 0.49 | 0.34 | 1 | 0.26 | 0.29 | 0.35 | 0.3 | 0.38 | 0.35 | 0.28 | 0.6 | 0.4 |
| t1_LEC | 0.42 | 0.31 | 0.32 | 0.34 | 0.46 | 0.33 | 0.26 | 1 | 0.48 | 0.25 | 0.36 | 0.37 | 0.47 | 0.33 | 0.24 | 0.42 |
| t2_CESD | 0.53 | 0.38 | 0.32 | 0.49 | 0.52 | 0.43 | 0.29 | 0.48 | 1 | 0.4 | 0.59 | 0.5 | 0.71 | 0.54 | 0.35 | 0.53 |
| t2_DAS | 0.3 | 0.5 | 0.2 | 0.34 | 0.35 | 0.31 | 0.35 | 0.25 | 0.4 | 1 | 0.32 | 0.3 | 0.39 | 0.39 | 0.35 | 0.3 |
| t2_CTI-C | 0.36 | 0.28 | 0.36 | 0.45 | 0.46 | 0.29 | 0.3 | 0.36 | 0.59 | 0.32 | 1 | 0.56 | 0.72 | 0.41 | 0.38 | 0.36 |
| t2_CNCEQ | 0.29 | 0.4 | 0.25 | 0.68 | 0.41 | 0.36 | 0.38 | 0.37 | 0.5 | 0.3 | 0.56 | 1 | 0.58 | 0.38 | 0.44 | 0.29 |
| t2_ATQ | 0.45 | 0.37 | 0.32 | 0.5 | 0.57 | 0.43 | 0.35 | 0.47 | 0.71 | 0.39 | 0.72 | 0.58 | 1 | 0.56 | 0.42 | 0.45 |
| t2_brooding | 0.34 | 0.38 | 0.18 | 0.38 | 0.4 | 0.57 | 0.28 | 0.33 | 0.54 | 0.39 | 0.41 | 0.38 | 0.56 | 1 | 0.31 | 0.34 |
| t2_ACSQ | 0.2 | 0.27 | 0.12 | 0.33 | 0.28 | 0.24 | 0.6 | 0.24 | 0.35 | 0.35 | 0.38 | 0.44 | 0.42 | 0.31 | 1 | 0.2 |
| t2_LEC | 0.29 | 0.26 | 0.22 | 0.36 | 0.37 | 0.26 | 0.19 | 0.54 | 0.49 | 0.19 | 0.46 | 0.46 | 0.52 | 0.31 | 0.25 | 0.29 |
| t3_CESD | 0.51 | 0.36 | 0.35 | 0.51 | 0.54 | 0.4 | 0.38 | 0.44 | 0.65 | 0.34 | 0.53 | 0.47 | 0.62 | 0.41 | 0.3 | 0.51 |
| t3_DAS | 0.27 | 0.45 | 0.22 | 0.34 | 0.35 | 0.26 | 0.32 | 0.17 | 0.36 | 0.59 | 0.3 | 0.31 | 0.37 | 0.31 | 0.34 | 0.27 |
| t3_CTI-C | 0.4 | 0.32 | 0.33 | 0.42 | 0.46 | 0.26 | 0.35 | 0.41 | 0.51 | 0.31 | 0.68 | 0.46 | 0.61 | 0.3 | 0.38 | 0.4 |
| t3_CNCEQ | 0.33 | 0.39 | 0.24 | 0.62 | 0.45 | 0.34 | 0.41 | 0.39 | 0.47 | 0.35 | 0.46 | 0.63 | 0.55 | 0.34 | 0.4 | 0.33 |
| t3_ATQ | 0.42 | 0.35 | 0.29 | 0.53 | 0.53 | 0.34 | 0.39 | 0.42 | 0.52 | 0.35 | 0.53 | 0.48 | 0.65 | 0.39 | 0.33 | 0.42 |
| t3_brooding | 0.37 | 0.26 | 0.23 | 0.35 | 0.4 | 0.45 | 0.22 | 0.29 | 0.46 | 0.25 | 0.33 | 0.32 | 0.47 | 0.5 | 0.22 | 0.37 |
| t3_ACSQ | 0.25 | 0.33 | 0.17 | 0.42 | 0.35 | 0.26 | 0.58 | 0.25 | 0.31 | 0.35 | 0.32 | 0.43 | 0.42 | 0.32 | 0.65 | 0.25 |
| t3_LEC | 0.25 | 0.21 | 0.16 | 0.38 | 0.31 | 0.18 | 0.18 | 0.51 | 0.42 | 0.27 | 0.39 | 0.35 | 0.45 | 0.28 | 0.23 | 0.25 |
| t4_CESD | 0.39 | 0.3 | 0.25 | 0.42 | 0.44 | 0.37 | 0.33 | 0.41 | 0.59 | 0.32 | 0.47 | 0.41 | 0.54 | 0.39 | 0.33 | 0.39 |
| t4_DAS | 0.19 | 0.45 | 0.16 | 0.31 | 0.27 | 0.28 | 0.25 | 0.21 | 0.27 | 0.51 | 0.21 | 0.29 | 0.28 | 0.28 | 0.27 | 0.19 |
| t4_CTI-C | 0.3 | 0.31 | 0.3 | 0.42 | 0.43 | 0.23 | 0.33 | 0.34 | 0.47 | 0.28 | 0.63 | 0.42 | 0.56 | 0.29 | 0.36 | 0.3 |
| t4_CNCEQ | 0.25 | 0.33 | 0.21 | 0.57 | 0.36 | 0.25 | 0.35 | 0.33 | 0.39 | 0.3 | 0.44 | 0.62 | 0.46 | 0.3 | 0.31 | 0.25 |
| t4_ATQ | 0.34 | 0.36 | 0.28 | 0.44 | 0.45 | 0.3 | 0.37 | 0.38 | 0.53 | 0.34 | 0.5 | 0.47 | 0.59 | 0.36 | 0.34 | 0.34 |
| t4_brooding | 0.38 | 0.31 | 0.27 | 0.35 | 0.42 | 0.5 | 0.28 | 0.29 | 0.42 | 0.31 | 0.36 | 0.37 | 0.47 | 0.46 | 0.2 | 0.38 |
| t4_ACSQ | 0.24 | 0.32 | 0.16 | 0.39 | 0.29 | 0.22 | 0.55 | 0.23 | 0.3 | 0.36 | 0.29 | 0.43 | 0.37 | 0.26 | 0.59 | 0.24 |
| t4_LEC | 0.23 | 0.16 | 0.19 | 0.33 | 0.29 | 0.18 | 0.18 | 0.47 | 0.39 | 0.19 | 0.43 | 0.28 | 0.41 | 0.22 | 0.21 | 0.23 |

**Table S1 (continued)**

| t3_CESD | t3_DAS | t3_CTI-C | t3_CNCEQ | t3_ATQ | t3_brooding | t3_ACSQ | t3_LEC | t4_CESD | t4_DAS | t4_CTI-C | t4_CNCEQ | t4_ATQ | t4_brooding | t4_ACSQ | t4_LEC |
| --- | --- | --- | --- | --- | --- | --- | --- | --- | --- | --- | --- | --- | --- | --- | --- |
| 0.29 | 0.51 | 0.27 | 0.4 | 0.33 | 0.42 | 0.37 | 0.25 | 0.25 | 0.39 | 0.19 | 0.3 | 0.25 | 0.34 | 0.38 | 0.24 |
| 0.26 | 0.36 | 0.45 | 0.32 | 0.39 | 0.35 | 0.26 | 0.33 | 0.21 | 0.3 | 0.45 | 0.31 | 0.33 | 0.36 | 0.31 | 0.32 |
| 0.22 | 0.35 | 0.22 | 0.33 | 0.24 | 0.29 | 0.23 | 0.17 | 0.16 | 0.25 | 0.16 | 0.3 | 0.21 | 0.28 | 0.27 | 0.16 |
| 0.36 | 0.51 | 0.34 | 0.42 | 0.62 | 0.53 | 0.35 | 0.42 | 0.38 | 0.42 | 0.31 | 0.42 | 0.57 | 0.44 | 0.35 | 0.39 |
| 0.37 | 0.54 | 0.35 | 0.46 | 0.45 | 0.53 | 0.4 | 0.35 | 0.31 | 0.44 | 0.27 | 0.43 | 0.36 | 0.45 | 0.42 | 0.29 |
| 0.26 | 0.4 | 0.26 | 0.26 | 0.34 | 0.34 | 0.45 | 0.26 | 0.18 | 0.37 | 0.28 | 0.23 | 0.25 | 0.3 | 0.5 | 0.22 |
| 0.19 | 0.38 | 0.32 | 0.35 | 0.41 | 0.39 | 0.22 | 0.58 | 0.18 | 0.33 | 0.25 | 0.33 | 0.35 | 0.37 | 0.28 | 0.55 |
| 0.54 | 0.44 | 0.17 | 0.41 | 0.39 | 0.42 | 0.29 | 0.25 | 0.51 | 0.41 | 0.21 | 0.34 | 0.33 | 0.38 | 0.29 | 0.23 |
| 0.49 | 0.65 | 0.36 | 0.51 | 0.47 | 0.52 | 0.46 | 0.31 | 0.42 | 0.59 | 0.27 | 0.47 | 0.39 | 0.53 | 0.42 | 0.3 |
| 0.19 | 0.34 | 0.59 | 0.31 | 0.35 | 0.35 | 0.25 | 0.35 | 0.27 | 0.32 | 0.51 | 0.28 | 0.3 | 0.34 | 0.31 | 0.36 |
| 0.46 | 0.53 | 0.3 | 0.68 | 0.46 | 0.53 | 0.33 | 0.32 | 0.39 | 0.47 | 0.21 | 0.63 | 0.44 | 0.5 | 0.36 | 0.29 |
| 0.46 | 0.47 | 0.31 | 0.46 | 0.63 | 0.48 | 0.32 | 0.43 | 0.35 | 0.41 | 0.29 | 0.42 | 0.62 | 0.47 | 0.37 | 0.43 |
| 0.52 | 0.62 | 0.37 | 0.61 | 0.55 | 0.65 | 0.47 | 0.42 | 0.45 | 0.54 | 0.28 | 0.56 | 0.46 | 0.59 | 0.47 | 0.37 |
| 0.31 | 0.41 | 0.31 | 0.3 | 0.34 | 0.39 | 0.5 | 0.32 | 0.28 | 0.39 | 0.28 | 0.29 | 0.3 | 0.36 | 0.46 | 0.26 |
| 0.25 | 0.3 | 0.34 | 0.38 | 0.4 | 0.33 | 0.22 | 0.65 | 0.23 | 0.33 | 0.27 | 0.36 | 0.31 | 0.34 | 0.2 | 0.59 |
| 1 | 0.42 | 0.2 | 0.44 | 0.37 | 0.44 | 0.28 | 0.25 | 0.57 | 0.43 | 0.23 | 0.47 | 0.36 | 0.43 | 0.33 | 0.2 |
| 0.42 | 1 | 0.41 | 0.65 | 0.57 | 0.71 | 0.5 | 0.4 | 0.43 | 0.62 | 0.3 | 0.51 | 0.47 | 0.54 | 0.45 | 0.37 |
| 0.2 | 0.41 | 1 | 0.39 | 0.4 | 0.46 | 0.34 | 0.4 | 0.25 | 0.31 | 0.59 | 0.36 | 0.41 | 0.42 | 0.36 | 0.38 |
| 0.44 | 0.65 | 0.39 | 1 | 0.56 | 0.71 | 0.34 | 0.41 | 0.41 | 0.43 | 0.18 | 0.62 | 0.42 | 0.51 | 0.35 | 0.33 |
| 0.37 | 0.57 | 0.4 | 0.56 | 1 | 0.67 | 0.43 | 0.51 | 0.43 | 0.44 | 0.35 | 0.48 | 0.71 | 0.53 | 0.36 | 0.45 |
| 0.44 | 0.71 | 0.46 | 0.71 | 0.67 | 1 | 0.5 | 0.51 | 0.52 | 0.52 | 0.3 | 0.57 | 0.5 | 0.62 | 0.44 | 0.37 |
| 0.28 | 0.5 | 0.34 | 0.34 | 0.43 | 0.5 | 1 | 0.31 | 0.31 | 0.44 | 0.28 | 0.3 | 0.34 | 0.43 | 0.5 | 0.29 |
| 0.25 | 0.4 | 0.4 | 0.41 | 0.51 | 0.51 | 0.31 | 1 | 0.26 | 0.35 | 0.32 | 0.38 | 0.43 | 0.39 | 0.31 | 0.69 |
| 0.57 | 0.43 | 0.25 | 0.41 | 0.43 | 0.52 | 0.31 | 0.26 | 1 | 0.44 | 0.25 | 0.45 | 0.37 | 0.43 | 0.32 | 0.23 |
| 0.43 | 0.62 | 0.31 | 0.43 | 0.44 | 0.52 | 0.44 | 0.35 | 0.44 | 1 | 0.35 | 0.62 | 0.46 | 0.68 | 0.5 | 0.41 |
| 0.23 | 0.3 | 0.59 | 0.18 | 0.35 | 0.3 | 0.28 | 0.32 | 0.25 | 0.35 | 1 | 0.32 | 0.36 | 0.44 | 0.45 | 0.41 |
| 0.47 | 0.51 | 0.36 | 0.62 | 0.48 | 0.57 | 0.3 | 0.38 | 0.45 | 0.62 | 0.32 | 1 | 0.59 | 0.7 | 0.4 | 0.38 |
| 0.36 | 0.47 | 0.41 | 0.42 | 0.71 | 0.5 | 0.34 | 0.43 | 0.37 | 0.46 | 0.36 | 0.59 | 1 | 0.59 | 0.43 | 0.45 |
| 0.43 | 0.54 | 0.42 | 0.51 | 0.53 | 0.62 | 0.43 | 0.39 | 0.43 | 0.68 | 0.44 | 0.7 | 0.59 | 1 | 0.57 | 0.47 |
| 0.33 | 0.45 | 0.36 | 0.35 | 0.36 | 0.44 | 0.5 | 0.31 | 0.32 | 0.5 | 0.45 | 0.4 | 0.43 | 0.57 | 1 | 0.35 |
| 0.2 | 0.37 | 0.38 | 0.33 | 0.45 | 0.37 | 0.29 | 0.69 | 0.23 | 0.41 | 0.41 | 0.38 | 0.45 | 0.47 | 0.35 | 1 |
| 0.55 | 0.37 | 0.23 | 0.38 | 0.36 | 0.41 | 0.28 | 0.18 | 0.67 | 0.46 | 0.18 | 0.56 | 0.46 | 0.48 | 0.28 | 0.2 |

*Note. CES-D*: Center for Epidemiological Studies – Depression scale; *DAS*: Dysfunctional Attitudes Scale; *CNCEQ*: Children’s Negative Cognitive Error Questionnaire; *CTI-C*: Cognitive Triad Inventory for Children; *ACSQ*: Adolescent Cognitive Style Questionnaire; *ATQ*: Automatic Thoughts Questionnaire; *Brooding*: RRS brooding subscale; *LEC*: Life Event Checklist.

**Table S2.** *Relative importance comparison based on 95% bootstrapping confidence intervals (5000 samples) across the four waves. Depressive symptoms (CES-D) served as outcome measure and cognitive vulnerabilities and life stressors as predictors.*

|  | (1) | (2) | (3) | (4) | (5) | (6) |
| --- | --- | --- | --- | --- | --- | --- |
| (1) DAS_Wave1 | - |  |  |  |  |  |
| (2) CTI-C_Wave1 | **12.94%;**  **21.79%** | - |  |  |  |  |
| (3) CNCEQ_Wave1 | -4.58%;  0.02% | **-23.51%;**  **-16.06%** | - |  |  |  |
| (4) ATQ_Wave1 | **20.13%;**  **18.39%** | -8.79%;  1.92% | **12.79%;**  **20.03%** | - |  |  |
| (5) Brooding_Wave1 | **0.23%;**  **6.88%** | **-18.59%;**  **-9.11%** | **2.95%;**  **8.84%** | **-15.04%;**  **-6.21%** | - |  |
| (6) ACSQ_Wave1 | -2.72%;  2.49% | **-21.83%;**  **-13.34%** | **0.02%;**  **4.34%** | **-18.28%;**  **-10.20%** | **-7.09%;**  **-0.33%** | - |
| (7) LEC_Wave1 | -0.92%;  5.75% | **-19.63%;**  **-10.51%** | **1.93%;**  **7.43%** | **-16.25%;**  **-7.50%** | -5%;  2.51% | -5.89%;  0.54% |

|  | (1) | (2) | (3) | (4) | (5) | (6) |
| --- | --- | --- | --- | --- | --- | --- |
| (1) DAS_Wave2 | - |  |  |  |  |  |
| (2) CTI-C_Wave2 | **2.12%;**  **10.68%** | - |  |  |  |  |
| (3) CNCEQ_Wave2 | -2.82%;  3.47% | **-10.15%;**  **-2.21%** | - |  |  |  |
| (4) ATQ_Wave2 | **6.50%;**  **15.11%** | **0.29%;**  **8.69%** | **6.45%;**  **14.43%** | - |  |  |
| (5) Brooding_Wave2 | **0.29%;**  **8.55%** | -7.19%;  3.05% | **0.30%;**  **7.82%** | **-10.89%;**  **-1.84%** | - |  |
| (6) ACSQ_Wave2 | -4.44%;  1.22% | **-12.04%;**  **-3.96%** | -4.61%;  0.65% | **-16.14%;**  **-8.17%** | **-9.70%;**  **-2.39%** | **-** |
| (7) LEC_Wave2 | **0.39%;**  **8.52%** | -6.62%;  3.28% | **0.25%;**  **8.09%** | **-10.37%;**  **-1.92%** | **-4.11%;**  **4.43%** | **-9.85%;**  **-2.48%** |

**Table S2 (continued)**

|  | (1) | (2) | (3) | (4) | (5) | (6) |
| --- | --- | --- | --- | --- | --- | --- |
| (1) DAS_Wave3 | - |  |  |  |  |  |
| (2) CTI-C_Wave3 | **4.08%;**  **13.32%** | - |  |  |  |  |
| (3) CNCEQ_Wave3 | -0.35%;  6.45% | **-10.71%;**  **-0.73%** | - |  |  |  |
| (4) ATQ_Wave3 | **6.91%;**  **14.02%** | -2.59%;  6.20% | **3.55%;**  **11.44%** | - |  |  |
| (5) Brooding_Wave3 | **0.63%;**  **7.94%** | -9.60%;  0.79% | -2.79%;  5.29% | **-10.19%;**  **-1.72%** | - |  |
| (6) ACSQ_Wave3 | -3.40%;  2.29% | **-13.95%;**  **-4.41%** | -6.56%;  0.30% | **-14.27%;**  **-7.47%** | **-8.62%;**  **-1.25%** | - |
| (7) LEC_Wave3 | -1.20%;  5.74% | **-11.40%;**  **-1.15%** | -4.61%;  3.13% | **-11.94%;**  **-4.03%** | -6.48%;  2.17% | -6.16%;  0.37% |

|  | (1) | (2) | (3) | (4) | (5) | (6) |
| --- | --- | --- | --- | --- | --- | --- |
| (1) DAS_Wave4 | - |  |  |  |  |  |
| (2) CTI-C_Wave4 | **3.76%;**  **13.19%** | - |  |  |  |  |
| (3) CNCEQ_Wave4 | -1.86%;  4.45% | **-11.90%;**  **-3.13%** | - |  |  |  |
| (4) ATQ_Wave4 | **5.31%;**  **13.33%** | -3.94%;  5.02% | **4.20%;**  **11.89%** | - |  |  |
| (5) Brooding_Wave4 | **1.98%;**  **9.71%** | -8.07%;  2.07% | **0.25%;**  **8.23%** | -8.45%;  0.68% | - |  |
| (6) ACSQ_Wave4 | -2.42%;  3.86% | **-12.81%;**  **-3.15%** | -3.89%;  2.68% | **-12.56%;**  **-4.87%** | **-9%;**  **-0.6%;** | - |
| (7) LEC_Wave4 | -0.70%;  7.08% | **-10%;**  **-0.83%** | -1.75%;  5.76% | **-10.35%;**  **-1.94%** | -6.67%;  1.83% | -6.39%;  1.27% |
| Note. DAS: Dysfunctional Attitudes Scale; CNCEQ: Children’s Negative Cognitive Error Questionnaire; CTI-C: Cognitive Triad Inventory for Children; ACSQ: Adolescent Cognitive Style Questionnaire; ATQ: Automatic Thoughts Questionnaire; Brooding: RRS brooding subscale; LEC: Life Event Checklist. | | | | | | |

**Figure S1.** *Representation of the correlation of the strength in the original network with the strength of the networks sampled while dropping participants, across the four waves.*

Wave 1

Wave 2

Wave 3

Wave 4

**Figure S2.** *Nonparametric bootstrapped confidence intervals of estimated edges for the four networks. The red line represents the estimated edge, while the dark area indicates the 95% boostrap confidence interval.*

Wave 1

Wave 2

Wave 3

Wave 4

**Figure S3.** *Nonparametric bootstrapped difference test for strength across the four waves. Gray boxes indicate no significant difference, whereas black boxes indicate statistically significant difference (p < 0.05). Diagonal values represent the strength score of each node.*

Wave 1

Wave 2

Wave 3

Wave 4

**Figure S4.** *Nonparametric bootstrapped difference test for edges, across the four waves. Gray boxes indicate no significant difference, whereas black boxes indicate statistically significant difference (p < 0.05). Diagonal color and saturation represent the magnitude and direction of each estimated edge.*

Wave 1

Wave 2

Wave 3

Wave 4

**Figure S5.** *Moderated networks across the four waves. LEC serves as moderator. Red squares represent the edge between nodes, while blue squares (not present) represent the moderating role of stressful events.*

**Figure S6.** *Network model across the four waves. Depressive symptoms are measured with the CDI.*

**Figure S7.**  *Strength scores across the four waves by different measure for depressive symptoms (CESD and CDI)*

**Figure S8.**  *Network model across the fours waves. CESD scores was computed after excluding the items overlapping with ATQ*

**Figure S9.** *Strength scores across the four waves. CESD scores was computed after excluding the items overlapping with ATQ*

**Figure S10.**  *Network model across the four waves, after controlling for age and gender*

**Figure S11.**  *Strength scores across the four waves, after controlling for age and gender*

**Figure S12.** *Network model in the original and imputed data sets across the fours waves.*

**

**Figure S13.** *Strength scores in the original and imputed data sets across the four waves*

**

**Figure S14.** *Moderated networks across the four waves. LEC serves as moderator. Red squares represent the edge between nodes, while blue squares (not present) represent the moderating role of stressful events.*

**
